# Takayasu's Arteritis and Crohn's Disease in a Young Hispanic Female

**DOI:** 10.1155/2014/246852

**Published:** 2014-07-24

**Authors:** Namrata Singh, Shireesh Saurabh, Irene J. Tan

**Affiliations:** ^1^Immunology, University of Iowa Hospitals and Clinics, 200 Hawkins Drive, C42 E 10, Iowa City, IA 52241, USA; ^2^Mercy Iowa City, 540 E Jefferson Street, Suite 205, Iowa City, IA 52245, USA; ^3^Section of Rheumatology, Temple University Hospital, 3200 North Broad Street, Philadelphia, PA 19140, USA

## Abstract

Takayasu's arteritis (TA) and Crohn's disease (CD) are chronic inflammatory granulomatous disorders of undetermined etiology. TA is a large vessel vasculitis with a predilection for the aorta and its branches in young women of Asian descent; whereas CD has characteristic gastrointestinal manifestations more prevalent in young Caucasians. We describe a case of both diseases in a young Hispanic female, review the literature, and impart new insight on possible genetic linkage and the role of interleukin 12 B (IL-12B) as the common autoimmune mechanism and potential therapeutic target in this rare disease combination.

## 1. Introduction

Takayasu's arteritis (TA) and Crohn's disease (CD) are chronic inflammatory granulomatous disorders of undetermined etiology. TA is a large vessel vasculitis with a predilection for the aorta and its branches; CD, on the other hand, has characteristic gastrointestinal manifestations [1]. TA is most commonly diagnosed in young women of Asian descent whereas CD appears to be more prevalent in young Caucasians.

Here, we report a case of both of these diseases in the same patient.

## 2. Case Report

A 26-year-old Hispanic female presented for evaluation of nausea, vomiting, abdominal pain, and watery diarrhea of two-week duration. She reported fevers, generalized malaise, and weight loss. She had similar gastrointestinal complaints in the past without any visual loss, weight loss, claudication, or joint pains. Her history was significant for appendicitis in 2003 and a perineal abscess/fistula repair in 2000. She denied smoking, alcohol, or illicit drug use.

**Figure 1:**
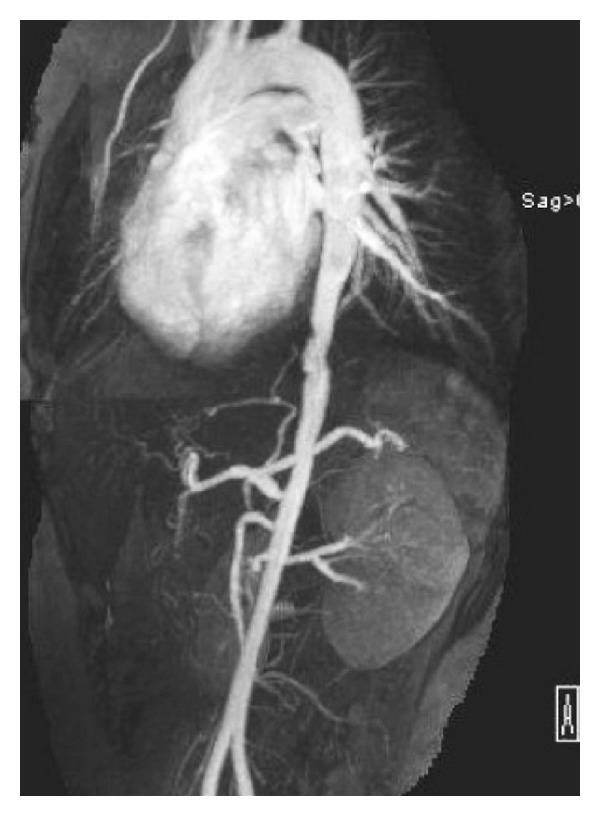
Magnetic resonance angiogram of chest and abdomen: severe irregularity and narrowing of the descending thoracic aorta. The subclavian arteries were not visualized in their entirety bilaterally during the initial imaging.

**Figure 2:**
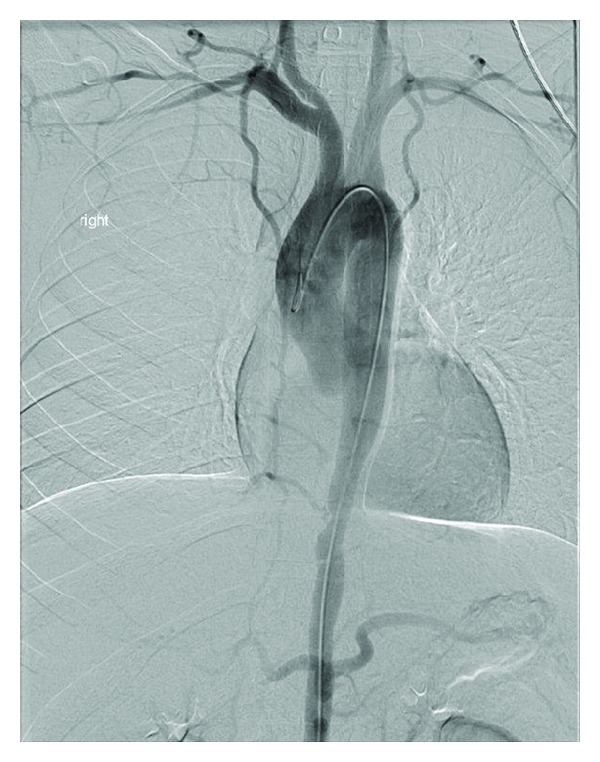
Follow-up thoracic and abdominal angiogram 14 months later: severe tapering of the subclavian arteries bilaterally, narrowing, and irregularity of distal aorta. The ascending aorta measured 2.5 cm whereas the distal thoracic aorta measured only 1.2 cm in diameter and tapered to 7.5 mm transversely at the narrowest point just beyond the aortic hiatus.

Physical examination revealed blood pressure of 126/73 mmHg in the right arm and 114/74 mmHg in the left arm. Her right radial pulse was not palpable and the left one was thready at 70 beats per minute. The femoral and dorsalis pedis pulses were difficult to appreciate bilaterally. An abdominal bruit was noted, as well as tenderness of the right lower quadrant. Musculoskeletal exam was normal except complaint of pain in both arms when raised above her head for more than 6 seconds. The shoulder exam was normal on both sides. Laboratory evaluation (Table 1) was significant for anemia of chronic disease and elevated inflammatory markers. She underwent colonoscopy and the right colon biopsy showed benign colonic mucosa with cryptitis, ulceration, and mild-moderate architectural change, consistent with Crohn's disease. Due to her symptoms of shoulder/arm pain, unequal blood pressure, and pulses in both arms and an abdominal bruit, further imaging studies were pursued. A magnetic resonance angiogram of the chest and abdomen was done and showed severe irregularity and narrowing of the descending thoracic aorta which tapers down from 2 cm transversely at the level of the distal arch to 7.5 mm transversely at the narrowest point just beyond the aortic hiatus. The subclavian arteries were not visualized in their entirety bilaterally during the initial imaging. There was 50% narrowing of the origin of the celiac artery with poststenotic dilatation; renal arteries were normal (Figure 1). At this point, the patient was diagnosed with Takayasu's arteritis based on the 1990 ACR criteria [2] in addition to Crohn's disease. The absence of recurrent oral and nasal ulcers and episodes of uveitis made Behcet's syndrome less likely. She had no hilar adenopathy; also her levels of serum calcium and angiotensin converting enzyme were normal, so sarcoidosis was low in the differentials. She was treated with pulse methylprednisolone for 3 days and started on adalimumab. A follow-up thoracic and abdominal aortogram 14 months later showed severe tapering of the subclavian arteries bilaterally and stable narrowing and irregularity of the distal aorta (Figure 2). Azathioprine has been added to her regimen; she is currently doing well and her steroids are being tapered.

## 3. Discussion

The association of TA and CD has been reported worldwide in fewer than 60 patients, mostly from Japan and Europe [3]. We found only 7 prior case reports from the USA [1, 4–9]. Although case reports of the association of Takayasu's arteritis and inflammatory bowel disease began in 1970 [10, 11], the first case of TA associated with CD was described by Yassinger et al. [4]. Kusunoki et al. [3] in 2011 listed 37 cases of individuals with this dual diagnosis found in the literature; of the 32 cases, whose age at diagnosis was noted, 25 cases (78%) had the onset of TA simultaneous with or later than that of CD. In their study of 44 patients with TA, Reny et al. [1] found CD to be present in 9% of the population and they reported that patients with coexistent TA and CD tend to be younger at the time of diagnosis and also tended to have systemic symptoms more frequently than those with TA alone. To our knowledge, no description of an association between anti-Saccharomyces cerevisiae antibodies (a serological marker of inflammatory bowel disease) and TA exists.

This association of TA and CD raises the question of whether it is more than coincidence to encounter these two different granulomatous diseases in the same patient. Interestingly, Maksimowicz-McKinnon and Hoffman [12] have pointed out the common features shared by these diseases despite their different clinical manifestations; both are diseases affecting young females and the pathogenesis of both includes predominantly TH1 lymphocytes and granulomatous inflammation.

Significant CD risk has been associated with genes like NOD2/CARD15, IBD5, and DLG5; IL23* R* has also been implicated as a CD susceptibility gene [13]. A pathway analysis using data from the Wellcome Trust Case Control Consortium (WTCCC) uncovered significant associations of CD and IL-12/IL-23 pathway components, harboring 20 genes such as IL12B, JAK2, STAT3, and CCR6 [14]. Glas et al. analyzed IL-12B gene variants regarding association with Crohn's disease and ulcerative colitis in German cohort and found that IL-12 single nucleotide polymorphism rs 6887695 modulates the susceptibility and the phenotype of inflammatory bowel disease [15]. IL-12 promotes the differentiation of naive CD4+ T cells into mature interferon-gamma producing Th1 effector cells and is a potent stimulus of natural killer and CD8+ T cells. In contrast, IL-23, a heterodimeric cytokine composed of a p19 subunit and a p40 subunit of which the latter is shared with IL-12, is required for the generation of memory T cells and drives differentiation of Th17 cells. A common genetic linkage between these diseases was not reported until recently when Terao et al. [16] performed genome scanning of 167 TA cases and 663 healthy controls via Illumina Infinium Human Exome Bead Chip arrays followed by a replication study consisting of 212 TA cases and 1,322 controls. They found that the IL12B (interleukin 12B) region on chromosome 5 and the MLX (Max-like protein X) region on chromosome 17 exhibited significant associations and the detection of these susceptibility loci will provide new insights to the basic mechanisms of TA pathogenesis. Their findings indicate that IL12B plays a fundamental role in the pathophysiology of TA in combination with HLA-B∗52:01 and that common autoimmune mechanisms underlie the pathology of TA and other autoimmune disorders such as psoriasis and inflammatory bowel diseases in which IL12B is involved as a genetic predisposing factor. Recently ustekinumab, a monoclonal antibody against IL-12 p40 subunit, was shown to be effective for patients with refractory CD [17]. The findings of Terao et al. raise the possibility of its therapeutic use for TA, and especially for patients with a dual diagnosis of TA and CD, by targeting the IL-12/23 pathway. Currently there are no guidelines on the management of patients with this dual diagnosis. There are case reports of TNF-inhibitor therapy being useful in these patients but few tolerated these biologic agents due to infectious complications. With further insight into the pathogenesis of these granulomatous diseases, we can hope for more effective and targeted therapies.

When providing care for a patient with a known diagnosis of an inflammatory bowel disease like CD, an awareness of the association with TA becomes very important especially because a delay in the diagnosis and then treatment of TA can have serious consequences for the patient including, but not limited to, congestive heart failure and cerebrovascular accidents. It is important for an internist to look for extraintestinal complications or Takayasu's arteritis in patients with IBD when the inflammatory markers are elevated out of proportion to patient's gastrointestinal complaints and their symptomatology/examination does not conform with their known medical problems.

## Figures and Tables

**Table 1:** Labs at the initial evaluation.

Lab	Patient's result	Normal range
Hemoglobin	10.5	11–15.9 g/dL
MCV	75	80–97 fL
WBC	5300	4000–10500/*μ*L
Platelets	359,000	140–415,000/*μ*L
BUN	9	6–20 mg/dL
Creatinine	0.54	0.57–1 mg/dL
AST	28	0–40 IU/L
ALT	40	0–40 IU/L
ESR	48	0–32 mm/hr
CRP	9	0–4.9 mg/L
Hepatitis panel	Negative	
Quantiferon	Negative	

WBC: white blood cells; MCV: mean corpuscular volume; mg/dL: milligrams per deciliter; BUN: blood urea nitrogen; AST: aspartate aminotransferase; ALT: alanine aminotransferase; IU: international units; ESR: erythrocyte sedimentation rate; CRP: C-reactive protein; and mm/hr: millimeters per hour.
